# Functional Mechanism of the Efflux Pumps Transcription Regulators From *Pseudomonas aeruginosa* Based on 3D Structures

**DOI:** 10.3389/fmolb.2018.00057

**Published:** 2018-06-19

**Authors:** Karim Housseini B Issa, Gilles Phan, Isabelle Broutin

**Affiliations:** Laboratoire de Cristallographie et RMN Biologiques (UMR 8015), Centre National de la Recherche Scientifique, Faculté de Pharmacie, Université Paris Descartes, Université Sorbonne Paris Cité, Paris, France

**Keywords:** multidrug resistance, efflux pumps regulators, activator, repressor, X-ray structures

## Abstract

Bacterial antibiotic resistance is a worldwide health problem that deserves important research attention in order to develop new therapeutic strategies. Recently, the World Health Organization (WHO) classified *Pseudomonas aeruginosa* as one of the priority bacteria for which new antibiotics are urgently needed. In this opportunistic pathogen, antibiotics efflux is one of the most prevalent mechanisms where the drug is efficiently expulsed through the cell-wall. This resistance mechanism is highly correlated to the expression level of efflux pumps of the resistance-nodulation-cell division (RND) family, which is finely tuned by gene regulators. Thus, it is worthwhile considering the efflux pump regulators of *P. aeruginosa* as promising therapeutical targets alternative. Several families of regulators have been identified, including activators and repressors that control the genetic expression of the pumps in response to an extracellular signal, such as the presence of the antibiotic or other environmental modifications. In this review, based on different crystallographic structures solved from archetypal bacteria, we will first focus on the molecular mechanism of the regulator families involved in the RND efflux pump expression in *P. aeruginosa*, which are TetR, LysR, MarR, AraC, and the two-components system (TCS). Finally, the regulators of known structure from *P. aeruginosa* will be presented.

## Introduction

Just after the introduction of antibiotics on the market in the mid-twentieth century, bacterial resistance was recognized as a natural but worrisome phenomenon (McDermott et al., 2003; Hede, 2014). More than 60 years later, the resistance is still a worldwide health concern (Frieri et al., 2017), threatening the effectiveness of antibacterial therapy, and challenging the efforts of developing novel antibiotics (Li et al., 2015), but fortunately some studies tend to give hope in this research area (D'Costa et al., 2011; Rolain et al., 2016).

To survive, bacteria have developed an inexhaustible range of antibiotic resistance mechanisms (Coates et al., 2002; Levy and Marshall, 2004). One of them involves the efflux of toxic compounds through bacterial cell-wall by membrane-bound protein transporters called multidrug efflux pumps (Poole and Srikumar, 2001; Rahman et al., 2017). These multidrug efflux systems (MES) existed in bacterial genomes long before the use of antibiotics by human to cure infection (Davies and Davies, 2010). MES are essential in bacterial physiology and natural defenses (Poole, 2008; Li and Nikaido, 2009; Alvarez-Ortega et al., 2013; Blanco et al., 2016), including export of organic solvent, detergents, fatty acids, toxic lipids and quorum sensing molecules. Because many structurally unrelated compounds are extruded by the same system, MES are also responsible for the multidrug resistance (MDR) phenotype (Nikaido, 2009). Efflux pumps have been categorized into five different families (Li and Nikaido, 2009), based on three criteria: the amino acid sequence identity, the energy source required to drive export and the substrate specificities (Li et al., 2015). The five major known families are the ATP-binding cassette (ABC) (Szakács et al., 2008; Locher, 2009), the small multidrug resistance (SMR) (Schuldiner, 2009), the major facilitator superfamily (MFS) (Kumar et al., 2013; Yan, 2015), the resistance-nodulation-cell division (RND) (Du et al., 2014; Yamaguchi et al., 2015; Daury et al., 2016; Vargiu et al., 2016) and the multidrug and toxic compound extrusion (MATE) (Hvorup et al., 2003; Kuroda and Tsuchiya, 2009). ABC superfamily belongs to the primary active transporters class which function depends on ATP hydrolysis, whereas the other pumps are secondary active transporters (symporters, antiporters, and uniporters) using energy from proton and/or sodium gradient (Mousa and Bruner, 2016).

Multidrug efflux transporters overexpression is tightly regulated by transcriptional activators and/or repressors upon the presence of toxic compounds (Sun et al., 2014). Interestingly, the regulators themselves are potentially triggered by the substrate that will be transported in turn by the regulated pumps (Schumacher and Brennan, 2002). A very specific and imbricated regulation system seems to link the transcriptional regulators to the cognate efflux pumps expression. In order to combat antibiotic resistance, all the different resistance mechanisms must be targeted, and despite recent encouraging results (Fair and Tor, 2014; Khameneh et al., 2016; Cheesman et al., 2017), multidrug transporters remain largely responsible for antibiotherapy failures (Sun et al., 2014). Because the last discovered antibiotic is specific to Gram positive (Gram+) bacteria (Ling et al., 2015), it is urgent to find new drugs targeting the Gram negative (Gram-). This is also supported by the fact that most of the main problematic multiresistant pathogens in hospitals or “ESKAPE” bacteria are Gram- (Tacconelli et al., 2017). This acronym comes from the initials of six superbugs which are *Enterococcus faecium* and *Staphylococcus aureus*, both Gram+, and the Gram- *Klebsiella pneumoniae, Acinetobacter baumannii, Pseudomonas aeruginosa and Enterobacter*. The particularity of Gram- bacteria is the presence of two membranes and a soluble space in between called the periplasm. Consequently, the efflux pumps must cross two lipidic barriers and the periplasm to transport the molecules out of the cell. This can be achieved by RND transporters that represent the major drug efflux pumps in Gram- bacteria, and more specifically by the hydrophobe/amphiphile efflux 1 (HAE1) sub-family (Nikaido, 2018) in which the transporter is a homotrimer that belongs to a tripartite complex. These efflux pumps are constituted of three different proteins forming an elongated nanomachine. The transporter itself, called RND, is localized in the inner membrane. It is the motor of the pump activated by the proton motive force. Another partner protein called Outer Membrane Factor (OMF) is embedded in the outer membrane. The third protein called Membrane Fusion Protein (MFP) is localized at the periplasm with a lipidic anchor inserted in the inner membrane. The 3D structure of the whole assembly has been solved recently by cryo-EM (Du et al., 2014; Daury et al., 2016; Wang Z. et al., 2017) highlighting how the different protein partners interact. Similar assembly architecture is also observed in the ABC family despite a different oligomeric organization of the transporter (Fitzpatrick et al., 2017). The fact that the HAE1-RND efflux system is only found in Gram- makes them interesting and specific target. Nevertheless, more than 10 years of research on this transporter family has not provided active and non-toxic drug yet. Thus, targeting the efflux pump expression regulation appears as an attractive alternative. Prokaryotic transcriptional regulators are classified into two groups: the one-component system and the two-components system. The gene expression regulation of the same HAE1-RND could involve both systems. A better comprehension of the molecular basis of efflux pumps genes expression is highly needed to pave the way for the design of new drugs toward multidrug resistance by efflux pump.

This review will focus on the regulators of the HAE1-RND efflux pumps (later called RND for simplification) involved in drug-resistance, with a particular focus on the regulator families controlling the pumps of *P. aeruginosa*, one of the most difficult bacteria to treat in clinic. For each family of the two-component and one-component regulators, a description of the molecular mechanism will be given based on structural knowledge obtained from archetypal organisms.

## The regulation outline of *P. aeruginosa* RND efflux pumps

**Figure 1:**
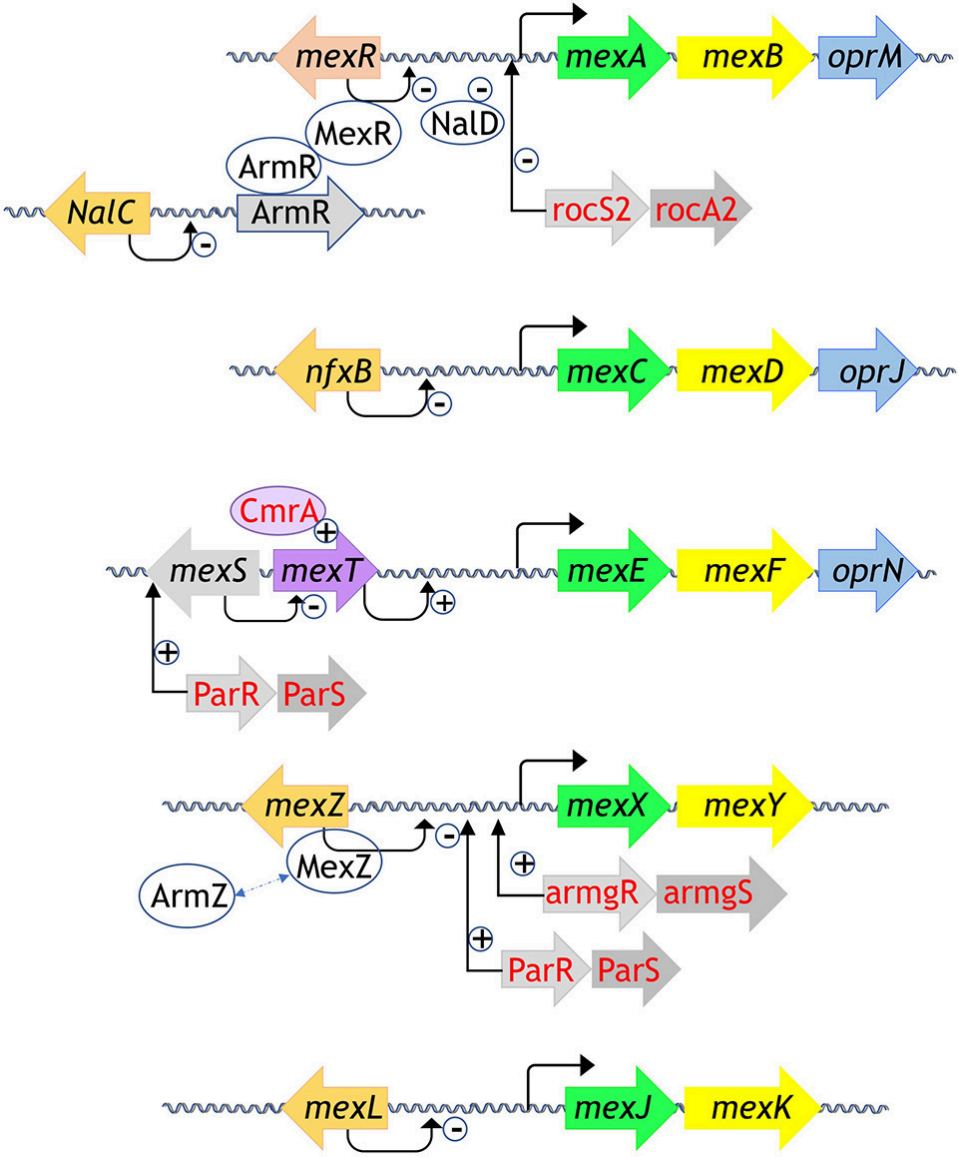
Regulation systems of the RND efflux pumps involved in antibiotic resistance from *P. aeruginosa*. Genes are schemed as arrows, proteins are oval shaped. The MFP are in yellow, the RND in green, the OMF in blue. The regulators from the TetR family are in orange, the MexR regulator from the MarR family is in salmon, MexT from the LysR family is in purple, CmrA coded by an AraC regulator is in light purple written in red, TCS are in gray written in red, all the other partners are in gray, written in black. Repression is indicated by “–“ sign and activation is indicated by “+” sign. All the 3D structures are generated with PyMol (http://www.pymol.org; DeLano, 2009).

*Pseudomonas aeruginosa* is an opportunistic bacterium that has the ability to rapidly grow in diverse environmental niches, from different soils to human respiratory tract. It is involved in severe human diseases like meningitis, septicaemia or cystic fibrosis and is also a major cause of nosocomial infections due to its high capacity to develop resistances (Poole, 2011). One of the most efficient resistance mechanisms is the overexpression of the tripartite RND-MFP-OMF efflux pumps. Up to twelve genes coding for the efflux pumps were identified in PAO1 genome, each of them showing substrates specificity (Stover et al., 2000). Nevertheless, only five of the efflux pumps are involved in resistance in clinical strains, i.e., MexA^MFP^-MexB^RND^-OprM^OMF^, MexX^MFP^-MexY^RND^-OprM^OMF^, MexC^MFP^-MexD^RND^-OprJ^OMF^, MexE^MFP^-MexF^RND^-OprN^OMF^, and MexJ^MFP^-MexK^RND^-OprM^OMF^ (Lister et al., 2009; Li et al., 2015). These tripartite pumps are encoded in operon, but some of them do not bear their own OMF gene, such as MexXY, MexJK, MexVW, and MexMN. In that case, they usually assemble with OprM, the versatile OMF of *P. aeruginosa*, which structure has been extensively studied (Phan et al., 2010, 2015; Monlezun et al., 2015). MexAB-OprM is the only pump that is constitutively expressed and is able to transport most of the antibiotic families whereas the others are more selective and are induced under specific conditions. Dedicated activators or repressors regulate the efflux pumps expression, but complementary regulators, including the two-components system, could intervene to finely orchestrate the operons transcription. A blast analysis (https://blast.ncbi.nlm.nih.gov/) of the regulators based on *P. aeruginosa* amino acid sequences and known 3D structure homologs allows us to identify the main families of regulators in *P. aeruginosa* as illustrated in Figure 1. A majority of them belongs to the TetR family. We will first describe the regulators function of each family from archetypal structures. We will start with the two-component system (TCS) and then the one-component systems, i.e., TetR, LysR, MarR, and AraC. Finally, we will present MexR, NalD and MexZ, the only 3D structures solved from *P. aeruginosa* one-component regulators so far.

## The two-component system regulators in *P. aeruginosa*

In bacteria, efficient adaptation to environmental changes is very often orchestrated by the two-component systems (TCS) (Stock et al., 2000). As such, TCS is one of the most abundant bacterial molecular devices to cope the variety of environmental signals (Krell et al., 2010; Capra and Laub, 2012; Jung et al., 2012). In particular, according to the whole genome prediction, *P. aeruginosa* owns around 130 different TCS (Rodrigue et al., 2000) and uses more than 60 TCS to regulate virulence and antibiotic resistance (Gooderham and Hancock, 2009; Muller et al., 2011; Li et al., 2015). All the TCSs regulating *P. aeruginosa* efflux pumps belong to the prototypical system, which mechanism will be described here.

Regarding the specific genes regulation of the RND efflux pumps, five TCSs were identified so far (see Figure 1 for those involved in resistance in clinical strains) (Li et al., 2015). The RocS2-RocA2 system was shown to downregulate the expression of the constitutive efflux pump MexAB-OprM in order to favor biofilm set up (Sivaneson et al., 2011). The TCSs ParR-ParS and AmgR-AmgS switch on the expression of the efflux pump MexXY following bacterial envelope stress and membrane perturbation by either colistin or polymyxin B (Fernández et al., 2010; Muller et al., 2011; Lau et al., 2015). Besides, ParR-ParS also upregulates the efflux pump MexEF-OprN operon by enhancing the expression of the activator MexS (Wang D. et al., 2013). Finally, both systems CzcR-CzcS and CopR-CopS stimulate the expression of the heavy-metal efflux pump CzcABC (Perron et al., 2004; Caille et al., 2007). This pump is not involved in antibiotic resistance but it is mentioned here as the only TCS crystal structure solved so far in *P. aeruginosa*. Indeed, the sensor domain of the zinc-responsive histidine kinase CzcS shows the typical mixed α/β-fold of the PhoQ family (Wang D. et al., 2017).

**Figure 2:**
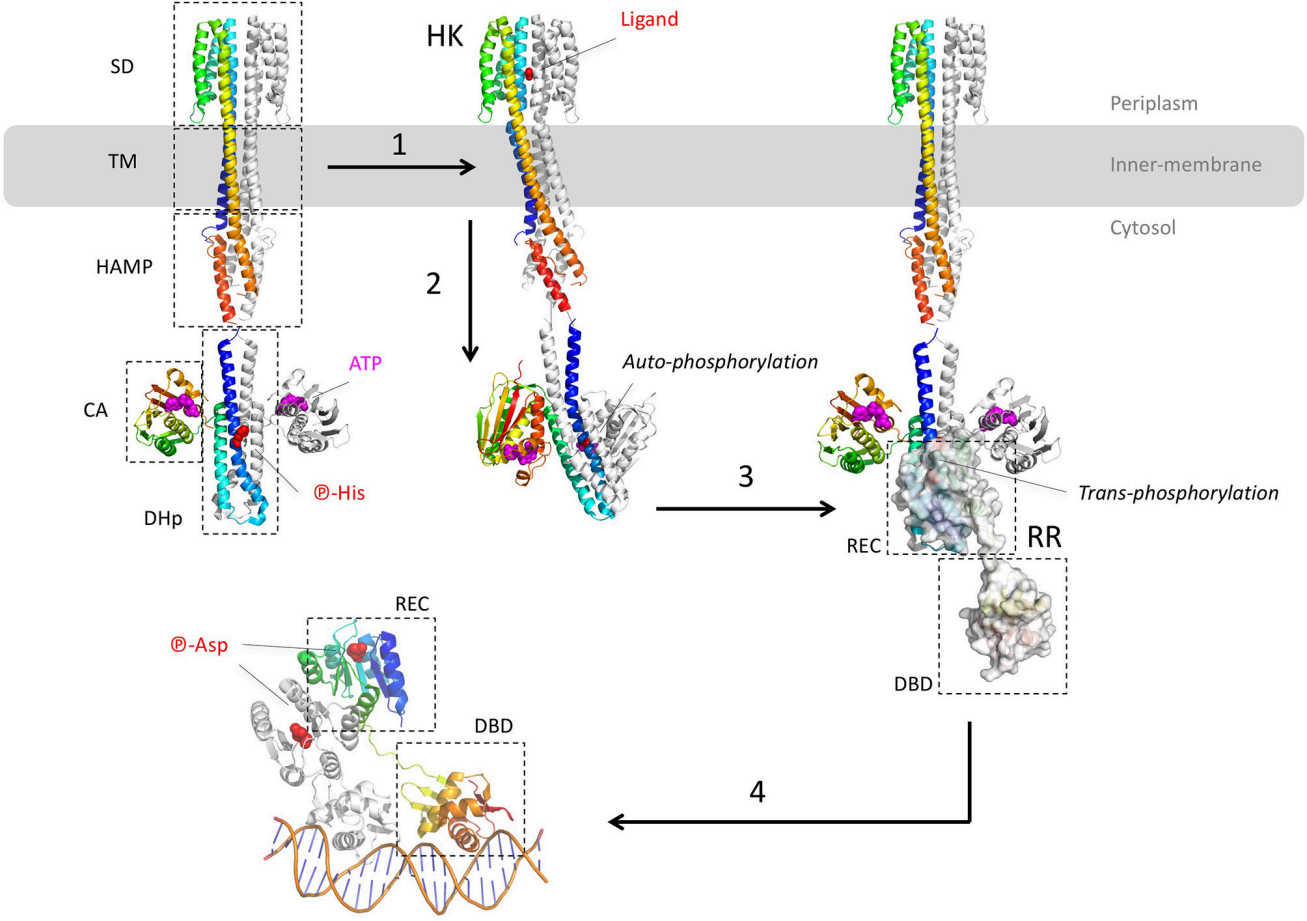
Structural overview of the two-component systems (TCS). The different model structures of histidine kinases (HK) are from the *E. coli* NarQ, CpxA, and HK853 (pdb codes 5JEQ, 5IEF, 4BIV, and 2C2A). The model structures of the response regulator (RR) are from the *E. coli* KdpE and RR468 (pdb codes 3DGE, 4KFC, and 4KNY). The different protein domains are indicated in dashed-squares: Sensor Domain (SD), TransMembrane domain (TM), transduction domain HAMP (*histidine kinases, adenyl cyclases, methylaccepting proteins, phosphatases)*, Catalytic Domain (CA), Dimerisation, and Histidine phosphotransfer domain (DHp), Receiver domain (REC), and Dinucleotidic acid Binding Domain (DBD). **(1)** After ligand binding to the p-helices (NO_3_ in the example of NarQ sensor kinase) of the symmetrical receptor homodimer, **(2)** important helices rearrangement forms an asymmetrical active state which triggers the auto-phosphorylation of DHp by CA. In this example, the CA of one monomer brings the ATP to the DHp of the other monomer. **(3)** The phosphor-histidine is then exposed to the REC domain of the response regulator (surface representation) that takes over the same phosphate following HK-RR complex formation. **(4)** Dimerisation of the aspartyl-phosphorylated RR allows DNA recognition by the DBD domain in a tandem manner, with the helix-turn-helix motif inside the major groove and the hairpin winged in the minor groove. All the 3D structures are generated with PyMol (http://www.pymol.org; DeLano, 2009).

Most of these TCS targeting the efflux pump genes of *P. aeruginosa* belong to the OmpR/PhoB family, except RocS2-RocA2, which is part of the CheY family. The architecture of the OmpR/PhoB and CheY families corresponds to the typical TCS which is a duet of phosphor-relay proteins (Figure 2): (1) a receptor Histidine-Kinase (HK), also named Sensor Kinase (SK), receives the extra-cytoplasmic (or periplasmic) signal and then activates (3) a cognate intracellular response regulator (RR) through a concerted trans-phosphorylation process (2). Subsequently, activated RR displays generally a DBD domain that targets a repeated sequence (4) upstream or within the promoter, in order to up-regulate or to repress the expression of specific genes (Krell et al., 2010; Capra and Laub, 2012; Zschiedrich et al., 2016).

Despite the lack of the full-length structure of HK, more than 100 structures of the different domains and fragments from different bacterial species shed light on the transduction mechanism upon ligand binding (Bhate et al., 2015; Zschiedrich et al., 2016). HK are membrane proteins and function as homodimeric receptors. The canonical monomer is made of 4 domains: (i) a sensor domain (SD) that recognizes various signals (gas, ions, osmotic change, temperature, light or variable organic compounds including antibiotics (Krell et al., 2010), (ii) a transmembrane domain (TM), (iii) one or several signal transduction domains (i.e., the domains HAMP - *histidine kinases, adenyl cyclases, methylaccepting proteins, phosphatases*, PAS—*per arnt sim* or GAF—*cGMP-specific phosphodiesterases, adenylyl cyclases, FhlA*) and (iv) an autokinase domain made of two subdomains DHp (*dimerization and histidine phosphotransfer)* and CA (*catalytic and ATP binding*). Thus, HK are multi-domains receptors with variable and complex architectures, but the structure of the transmembrane and the cytosolic domains tend to be more conserved than the sensor domains across the receptor family (Krell et al., 2010; Bhate et al., 2015; Zschiedrich et al., 2016).

The different domains of HK show diverse folds, mainly from α/β to all-α classes. Behind the variety of stimuli and domain structures, signal transduction mechanism tends to be well conserved, driven by key α-helices connecting the different domains along the receptor. Signal transduction mechanism starts at the sensor domain by the p-helices (*periplasmic helices*) which are localized at the dimer interface of the sensor domain. Upon binding of the ligand, helices rearrangements, described as a piston-like shift, are transmitted to the TM domain across a bundle of two pairs of anti-parallel α-helices connected to the cytosolic domain, mainly HAMP, PAS, or GAF domains. As for the sensor domain, cytosolic domain folds are variable but the signal transduction is again driven by specific α-helices, in particular a tandem of input and output helices. Through dynamic scissoring and rotation of the α-helices bundle, a major structural event is the transition from a symmetrical apo-conformation (ligand-free) to an asymmetric holo-conformation (ligand-bound) and then active state. To summarize the complex signal transduction within the receptor structure, a global α-helices coiled-coil disturbance mediated by the key helices stretching and rotation leads to an asymmetric kinase-competent state (Wang C. et al., 2013; Mechaly et al., 2014; Molnar et al., 2014; Gushchin et al., 2017).

The catalytic event of HK takes place at the DHp and CA domains. It starts with the auto-phosphorylation of the conserved histidine of DHp domain which is a symmetrical dimer of helix-turn-helix. The CA domain binds to the upper region of the DHp and captures the phosphoryl group from one ATP to phosphorylate the histidine of DHp. Depending of the orientation of the helices, histidine phosphorylation could be within the same protomer (*cis*-phosphorylation) or between (*trans*-phosphorylation) the subunits of the homodimer receptor (Casino et al., 2009, 2010). The lower part of DHp receives the response regulator RR. Again, a critical switch to an asymmetric conformation of the DHp correlates with the active kinase state. The formed phosphor-histidine is then available for the trans-phosphorylation to the RR.

Actually, the RR protein is a kinase itself and catalyzes its own phosphorylation on a conserved aspartate. The prototypical RR of the OmpR/PhoB superfamily presents two domains: a conserved N-terminal receiver (REC) domain linked to a more variable C-terminal domain, mainly a DBD. The typical REC domain consists of a typical α/β fold with five parallel β-strands surrounded by five α-helices. The REC domain docks onto the lower part of the DHp domain and catalyzes its own aspartyl-phosphorylation from the phospho-histidine donor. This phosphor-relay disturbs the molecular surface of REC and triggered the switch of two conserved residues T/S and F/Y localized between the strands β4-β5 of REC, nearby the phosphor-aspartate. This event induces the symmetric dimerization of the REC domain (Gao and Stock, 2009) and brings in close proximity the C-terminal DBDs each other. Thus, the DBD tandem is able to recognize and to interact with the DNA repeat sequence. Remarkably both REC domains of the RR dimer form a symmetrical head-to-head complex, whereas the associated DBD are poised asymmetrically on the cognate DNA, in a head-to-tail manner (Narayanan et al., 2014; Lou et al., 2015; He et al., 2016). Structural determination of the DNA-RR complexes revealed that the DBD-DNA interface is conserved, described as a winged-helix fold where the recognition α3 penetrates the major groove whereas the α-β hairpin wings interacts with the minor groove, motif found in the LysR and MarR of the one-component system (see below) (Blanco et al., 2002; Narayanan et al., 2014; Lou et al., 2015; He et al., 2016). The surface contact of the DBD-DNA complex covers around 1,800 Å^2^ with mainly van der Waals interactions toward the ribose groups and electrostatic attractions to the phosphates backbone.

## The one-component system regulators

The one-component regulator system comprises both activators and repressors depending on the location of their binding site (TFBS) with the one of the RNA polymerase (RNAP). If the TFBS interferes with RNAP binding, the transcription factor will act as a repressor. When located upstream, the transcription factor helps RNAP recruitment as an activator or by competing with a repressor. One-component regulators can either act locally, interfering directly with the regulated operon, or remotely through general signaling events. These regulatory proteins are composed of two domains, one DNA-binding domain (DBD) comprising a Helix-Turn-Helix (HTH) motif, and one sensory domain involved in the oligomerization of the protein, often triggered by the binding of the sensor molecule. The one-component regulators are implicated in most of the essential signaling events in prokaryotic cells that is why a majority of bacterial regulators belong to this system. They are classified into more than 20 families (Cuthbertson and Nodwell, 2013) mainly based on sequence similarity of the DBD (Grkovic et al., 2002), and most of them belong to six major families: TetR, MarR, LacI, LysR, AraC and MerR (Spengler et al., 2017). Four of them are involved in *P. aeruginosa* RND efflux pumps regulation (TetR, MarR, LysR, AraC) and they will be described here.

### The TetR family

The tremendous amount of sequences deposited in the UniProtKB databank (>2,300 verified TetR family assigned sequences in 2005; Ramos et al., 2005) supports the fact that most of bacterial genomes carries several TetR regulators to control vital and diverse functions. Interestingly, more than 15% of them regulate membrane-associated proteins, which are transporters in majority. As expected, the number of solved structures deposited in the protein data bank (PDB) is still low with hardly more than 280 entries (December 2017). The first solved 3D structure of a member of this family corresponds to the tetracycline repressor (TetR) from *E. coli* [2TCT (Kisker et al., 1995); 2TRT (Hinrichs et al., 1994)] repressing the expression of TetA, a MFS efflux pump expulsing the tetracycline antibiotic. Surprisingly, structural alignment of the known structures of this family did not provide conserved motif because of a low sequence identity (as low as 7%), despite the well conserved fold of the N-terminal DBD domain (≈50 amino acids). Nevertheless, this analysis highlights the fact that most of the studied regulators are frequently involved in antibiotic resistance or virulence of pathogenic bacteria. The larger group of solved structures corresponds to EthR from *Mycobacterium tuberculosis* with 57 entries (Carette et al., 2012; Blondiaux et al., 2017; Nikiforov et al., 2017). It negatively regulates the expression of EthA monooxygenase implicated in the inactivation of the anti-tuberculosis drug ethionamide.

**Figure 3:**
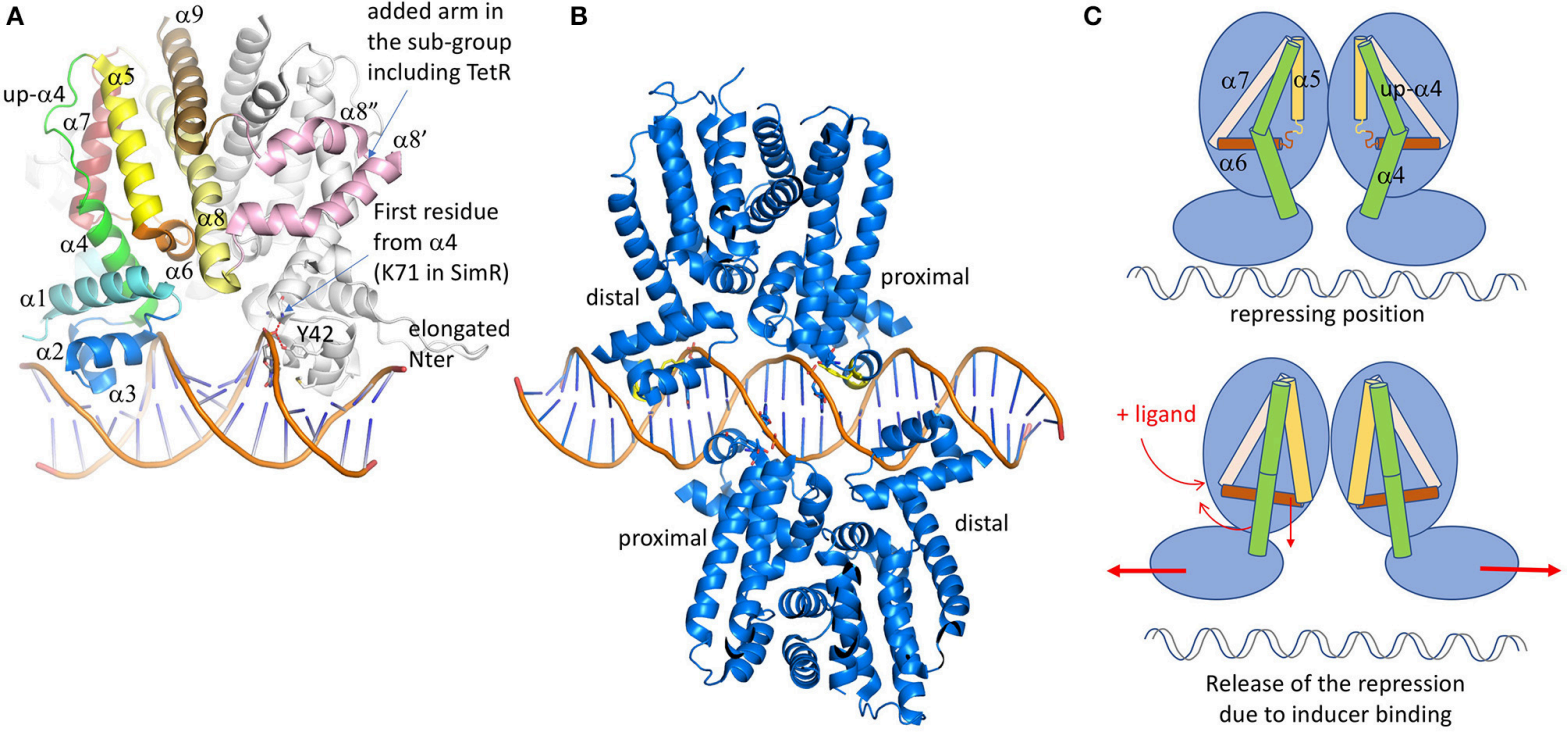
TetR family of regulators. **(A)** Structure of SimR (PDB code 3ZQL) from *Streptomyces antibioticus*, presenting an elongated N-terminus in comparison with the classical TetR regulators and two additional α helices (in pink) in between the dimer interface α8 and α9 helices (light yellow and brown). The inducer binding triangle (α5–α7) is colored yellow, brick and salmon. The DBD domain [α1 plus the HTH (α2 and α3)] is colored blue. Helix α4, transmitting the conformational modifications from LBD to DBD is colored in green. In several structures, the upper part of α4 is unfolded and adopts a helical structure after ligand binding. **(B)** Structure of QacR from *S. aureus* (PDB code 1JT0) representing the sub-group of the TetR regulators, acts as a dimer of dimers interacting in antiparallel manner on the 5′-CTATA-n9-TATAG-3′ DNA pseudo-palindromic fragment. The two monomers near the center of the DNA sequence are called proximal monomers and the other two are called distal monomers. (C) Schematic representation of the TetR regulation mechanism. When the ligand binds to the LBD, the enlarged triangle cavity pushes α5 and α6, which induces the folding of the helices extremities. As a consequence, α4 unbends to create a linear helix. The induced movement pushes away the two DBD domains, releasing the regulator from the DNA fragment. All the 3D structures are generated with PyMol (http://www.pymol.org; DeLano, 2009).

In spite of low sequence identity, the 3D structure of these regulators is conserved (Figure 3A). It is formed of 9 α-helices, with α1-α3 corresponding to the DBD and α4- α9 to the ligand-binding domain (LBD) (Deng et al., 2013). The contacts between the two domains involve α1 with α4 and α6, the latter being perpendicular to the dimer interface formed by α8 and α9 of each subunit (Figure 3). These inter-domain helices play a key role in the regulator activation through conformational modifications induced by the ligand binding. Among the 280 structures of TetR deposited in the PDB, more than 80 are declared to be associated with ligands. The chemical nature of these ligands is diverse, from a simple benzenediol such as resorcinol interacting with RolR, regulator of the aromatic catabolism from *Corynebacterium glutamicum* (3AQT, Li et al., 2011), to a more complex nucleotide derivative compound like isovaleryl coenzyme A (IV-CoA), an important building block in the formation of iso-fatty acids, interacting with AibR from *Myxococcus xanthus* (5K7H, 5K7Z; Bock et al., 2017). Most of the regulators classically repress the transcription in their apo-form, except for the AibR which is a repressor in a ligand-bound form of a whole operon bearing five genes involved in the biosynthesis process of IV-CoA, once bound to the same ligand. Besides, protein partners instead of chemicals also modulate some regulators. For instance, the SlmA regulator involved in cell-division of *E. coli* (Schumacher and Zeng, 2016) or AmtR, the nitrogen regulator of *Corynebacterium glutamicum* (Palanca and Rubio, 2016), both have to bind to DNA and another cognate protein at the same time to act. The diversity of the TetR regulator/ligand complexes is not limited to the ligand nature, but also depends on the localization of the binding regions which could be at the protein surface, close to the dimer or the domain interfaces, or in a deeply buried cavity that could even cross the entire protein like ActR (see Figure 8 from Cuthbertson and Nodwell, 2013 for a graphical representation). It differs also by the stoichiometry of the binding molecules, perfectly exemplified with TtgR (Alguel et al., 2007), a repressor of the key efflux pump TtgABC in *Pseudomonas putida*. Unlike TetR, which is only activated by tetracycline, TtgR can accommodate different molecules in the same ligand-binding site. Five structures have been solved of TtgR in complex with different antimicrobial molecules (2UXH, 2UXI, 2UXO, 2UXP, and 2UXU). In three different structures, the ligand occupies a large cavity formed by helices α5-α8, within each monomer (quercetin in 2UXH, chloramphenicol in 2UXP and naringenin in 2UXU). In the complex with tetracycline (2UXO), a single monomer site is occupied despite the structural similarity with quercetin or naringenin. On the contrary, the structure of TtgR with phloretin shows both cavities occupied with an additional molecule close to α6 but in one monomer only. Using isothermal calorimetry to measure the binding of the different molecules, it has been shown that the affinity of phloretin for the different binding sites differs by two orders of magnitude, suggesting the existence of a positive cooperativity between the two sites.

Because of the important diversity of the ligands and the binding sites, it will not be possible to bring out a prototypical interaction mechanism. Nevertheless, the global structural analysis of the LBD shows a conserved helical architecture built around helices α5–α7 forming the so-called triangle that can be superimposed easily despite a root-mean-square deviation (rmsd) of more than 15 Å. This long atomic distance is due to differences in helices length and curvature. α8 is parallel to α5, stabilizing the triangle. Even if most of LBD domains are formed by 6 helices (α4 to α9), TetR belongs to a subclass itself, together with some minority proteins like the ActR regulator of actinorhodin efflux pump from *Streptomyces coelicolor* (Willems et al., 2008). We notice that the family name TetR was historically given because it was the first structure solved, but it is actually not representative since the folds are quite variable. For instance, in a sub-class of the TetR family, two additional helices are inserted between the classical α8 and α9, keeping the C-terminal helix at the conserved dimer interface. The two extra helices adopt a coiled-coil structure forming an arm that shell the second monomer, thus stabilizing the dimer. This is not the only difference in this sub-family. They also present a shorter helix α4 at the interface between the LBD and the DBD, the upper part of the traditionally curved α4 being unstructured but still parallel to α5.

In all the TetR family regulators, the dimer interface is formed by two antiparallel coiled-coil helices from each monomer (α8 and α9), forming a symmetrical four-helices bundle with the other monomer. The dimer is not always symmetrical, that is why ligands are not always present in both cavities of the complex structures. This is the case of QacR (Schumacher et al., 2004), which presents a much smaller cavity in one of the two monomers due to the flexibility of the last turn of α5, giving more freedom to α6 that can move upper then reducing the triangle cavity. The binding of the ligand in only one cavity is sufficient to release the transcription repression, but two dimers of QacR must interact with DNA (Schumacher et al., 2002), so at the end two ligand-bound cavities are necessary for the DNA recognition. In QacR the enlarged cavity can accept two different molecules (ethidium and proflavin), even if proflavin displaces ethidium from the binding site. Ligand binding pushes α6 in an allosteric way, which is transmitted to α4 that adopts a pendulum-like movement as described in Resch et al. (2008), driving away the two DNA binding sites (Figure 3C). It has also been described in HrtR, the heme homeostasis regulator in *Lactococcus lactis* (Sawai et al., 2012), an induced structural modification of this α4 from a partial random-coil to stable α-helical structure once associated to the cognate DNA. In the case of QacR, the distance between the two sites increases from 37 to 45 Å, causing the detachment of the regulator from the DNA fragment (Schumacher et al., 2001). It has been suggested by Reichheld et al. (2009) that the rigidification of the structure drives the DNA release. This hypothesis is based on far-UV circular dichroism experiments on TetR wild-type and mutants in the presence of increasing concentration of urea, with and without ligands. In the absence of the ligand, the DBD unfolds first followed by the LBD. In presence of the ligand, the two domains unfold at the same time, suggesting cooperativity. The instability of DBD domain without the ligand would be a clue for its DNA adaptation.

The DBD, generally localized at the N-terminus of the protein, is composed of α1, HTH domain, and the beginning of α4. Among the 280 deposited TetR structures, only 19 are associated with the cognate DNA fragments, highlighting the difficulty to stabilize these protein/DNA complexes. These regulator-DNA complex structures were crucial to understand the functional mechanism of this regulator family. There are two sub-classes of DNA-binding mode: one represents DNA promoters interacting with a dimer (1QPI: TetR; 3ZQL: SimR; 3LSP and 3LSR: DesT; 3VOK: HrtR; 5UA2 and 5UA1: KstR; 5DY0: AmtR; 4I6Z: Tm1030; 5K7Z: AibR). The second involves two dimers interaction (1JT0: QacR; 5HAW, 5HBU and 5k58: SlmA; 4PXI and 5H58: CprB; 2YVH: CgmR; 4JL3: Ms6564; 5GPC: FadR) (Figure 3B). The first DNA complex was determined 5 years after the first structures of TetR by the same research group (1QPI; Orth et al., 2000). The main interacting region corresponds to the HTH domain formed by α2 and α3, which is also the most conserved sequence region used to create an identification profile of the TetR family (Ramos et al., 2005). The two helices deeply enter the DNA major groove but most of the contacts involve α3 only. In DesT, a regulator that controls the fatty acid saturation ratio in membrane lipid biogenesis, additional interactions were described with the DNA minor groove involving the elongated N-terminus of the protein (Miller et al., 2010). This is also the case for SimR, an exporter of a potent DNA gyrase inhibitor from *Streptomyces antibioticus*, which presents an even longer N-terminus that turns back to the added “arm” between α8 and α9 of the second monomer (Le et al., 2011). CprB from *S. coelicolor*, a receptor of c-butyrolactones, a class of quorum sensing molecules (Bhukya et al., 2017), also takes part of this N-terminal extended sub-family. AmtR, the global nitrogen regulator of *C. glutamicum* that is activated by a protein instead of a small molecule (Palanca and Rubio, 2016), also shows an additional C-terminal helix of unclear function. The recognized DNA operator is often formed by one central base pair (bp) surrounded by a palindromic sequence of a minimum of 6 bp in each opposite direction from the center (Yu et al., 2010). So, the complex is formed by a symmetrical protein dimer bound to a symmetric DNA fragment. Among the sub-class acting as a dimer of dimers, the regulators bind two overlapping DNA palindromic sequences instead of one in a cooperative manner. It is the case of QacR (Grkovic et al., 2001; Schumacher et al., 2002), which represses the expression of the MFS efflux pump QacA, transporting toxic organic compounds like the *q*uaternary *a*mmonium *c*ompounds. In this case, the palindromic DNA fragment is elongated by 28 bp with a longer central non-palindromic sequence (6 instead of 1) in order to accommodate the two dimers. For this class of regulators, the following nomenclature has been adopted: depending on the distance of the dimer from the central DNA sequence, there are the proximal and the distal subunits. Both dimers bind on opposite side of the promoter sequence, the two proximal monomers being very close to each other, sharing several DNA base pairs in their binding site. With the opposite binding mode, the two dimers axis form an angle of <180° except Ms6564 (Yang et al., 2013), a broad regulator in *Mycobacterium smegmatis* that presents a smooth interaction involving water molecules which allow a sliding motion of the regulator along the genome to target several genes. For instance, the measured angles are 130° for both QacR and SlmA (the smallest ones), 142° for CprB and 145° for CgmR (Itou et al., 2010; Bhukya et al., 2014). In spite of this particularity, the interaction of two dimers with the DNA is very similar to that of one dimer. The main difference is a larger spacing between the two DNA binding sites within a dimer (3 bp for TetR-like regulators and 4 bp for QacR-like), reducing the induced bending of the DNA fragment (≈3° compared to ≈16° for the one-dimer class) and increasing the α3-α3' distance from ≈34 to ≈37 Å. This distance is comparable to one turn of DNA-*B* form and was questionable for a long time. The numerous unbound and ligand-bound structures show very variable distances between these two α3 helices, ranging from 37.4 to 48.1 Å (Yu et al., 2010). This was confusing for scientists in their interpretation of the regulation mechanism, as discussed by Frénois et al. (2004) when they compared the structure of EthR solved with hexadecyl octanoate in LBD, and that of QacR solved in different forms (free, complexed with a ligand and complexed with DNA).

When superposing the DBD from the solved TetR structures in complex with DNA, it clearly appears that the HTH and the N-terminus of α4 of the DNA binding site match perfectly without any amino acid insertion. There are eight important residues for the binding: the two first correspond to the first residues of α2 and α4 that are not conserved since the interaction involve the protein backbone only with the phosphates of the DNA. The six other residues correspond to the whole α3 helix except the central residue oriented toward α2 (T41 in TetR). The nature of these amino acids varies with the cognate DNA sequence, even though most of the protein/DNA interactions involve the DNA backbone and not the nucleic base. This is the case for AibR presenting seven residues interacting with phosphates backbone and only two involving specific contacts with the base (Bock et al., 2017). Nevertheless, one residue is particularly well conserved, a tyrosine (Y42 in TetR and Y40 in QacR) that interacts with the same DNA phosphate as the amine group of the N-terminal residue of α4, reinforcing the DNA binding. This phosphate corresponds to the center of the DNA palindromic sequence. The amino acid the most deeply buried into the DNA major groove is positioned four residues before the conserved tyrosine (Y-4), in the turn preceding helix α3. This amino acid is often a short one (Ramos et al., 2005). In the case of FadR, a regulator involved in the fatty-acid degradation and synthesis pathways, mutation of each of these two conserved residues led to a significant reduction of DNA binding as proved by electrophoretic mobility shift assays (Yeo et al., 2017). It seems that the regulator could slide on the DNA until the α3 helix recognizes the cognate sequence, using a short residue at position Y-4 to enter more deeply into the groove. Then the clamp formed by the tyrosine and the first residue of α4 lock the interaction.

### The LysR family

**Figure 4:**
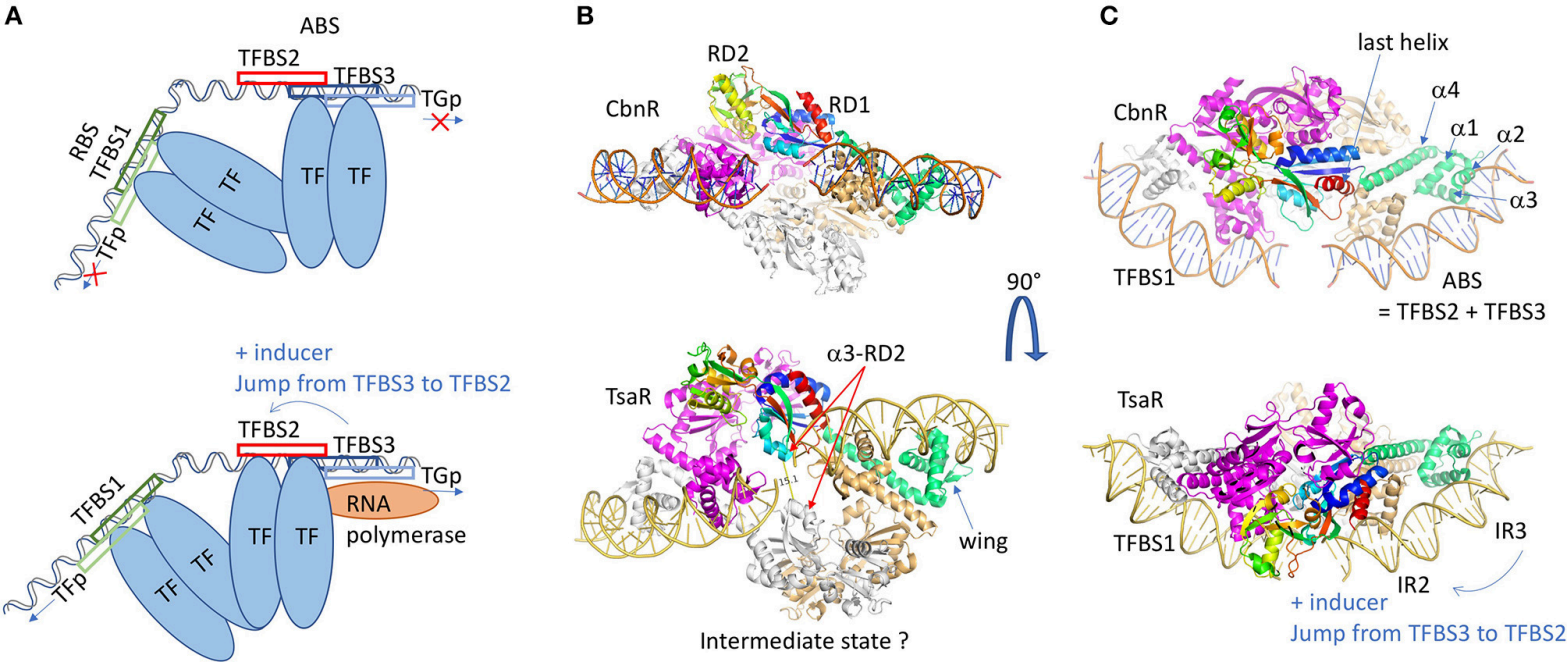
LysR family of regulators. **(A)** Schematic representation of the LysR regulation mechanism. Two dimers of transcription factors are involved in the regulation and bind at two different sites on a long DNA fragment (usually 75 bp), the RBS and the ABS. The RBS contains TFBS1, the transcription-binding site of higher affinity, and the promoter sequence of the LysR. The ABS is usually composed of TFBS2 and TFBS3, overlapping with the target gene promoter (TGp). In the repression state, the second dimer binds to the TFBS3, and the dimer of dimers of TF imposes a high bending of the DNA fragment. In presence of the inducer, the dimer of dimers undergoes conformational changes leading to its sliding from TFBS3 to TFBS2, which releases the DNA bending. This gives access of the TGp to the RNAP and the TG transcription can start. **(B)** Structure representation of the dimer of dimers of CbnR (1IZ1: upper view) and TsarR (3FZJ: bottom view). In order to visualize the DNA placing the structure of the DBD/DNA complex of CbnR (5XXP) is superposed on the DBD of each proteins' monomer. One dimer is colored gray and magenta. In the second dimer, one monomer is light brown and the other monomer is colored by domains. The DBD is in green. The RD is in rainbow. The TsaR structure would correspond to an intermediate state, as its RD domains are in a very different conformation compared to CbnR. There is an increase of the distance between the two helices α3 from RD2 (α3-RD2 on the figure), a concomitant decrease distance of the two HTH dimers and a kink of the DNA fragment. This could mimic the intermediate state before the TF jump **(C)** is a perpendicular view of **(B)** All the 3D structures are generated with PyMol (http://www.pymol.org; DeLano, 2009).

The transcription factors (TF) belonging to the LysR family (LysR-Type Transcription Regulators: LTTR) are the most abundant in prokaryotes. This is due to the fact they regulate the expression of genes coding for proteins involved in very diverse functions like β-lactamase, transporter, amino acids biosynthesis, metabolic signaling, secretion, oxidative-stress response, cell division, quorum sensing, virulence, motility, detoxification, attachment (Schell, 1993; Maddocks and Oyston, 2008; Jiang et al., 2018). The family was named after the extensively studied transcription regulator of *lysA* implicated in lysine biosynthesis (Stragier et al., 1983) and is composed of both activators and repressors (Maddocks and Oyston, 2008) depending on the location of the transcription factor binding site (TFBS). The genetic organization of LTTRs targeted promoters and TFBSs has been studied by a computational protocol termed Phylogenetic Profile of Consensus Motifs issued by the analysis of Phylogenetic Footprinting technics (Oliver et al., 2016). In the LysR family, the gene coding for the TF is divergently oriented from its target gene (TG) and located <100 nucleotides upstream the beginning of the TG, and sometimes up to 500 bp from the initiation codon (Heroven and Dersch, 2006). Two to three different TFBSs are found in the intergenic region, the inter-motif length between the two first is generally seven nucleotides, except for the LyrR which is six. When the third motif exists, it very often overlaps with the second one, the global site being called the ABS (activation binding site) (Figure 4A). LysR also differs for this rule, because it presents 19 bp intermotif length between TFBSs 2 and 3. In each case, the transcription activation involves the binding, with different affinities, of two activated TF dimers in a cooperative manner, triggered by one or several inducers. An overlap of TF promoter and TFBSs causes an auto-repression by the TF when bound to the TFBS on the opposite strand of the DNA. The global site is called the RBS (regulatory binding site). Depending on the affinity of the TF for one of the different TFBSs, the TF will be an activator or a repressor. The mean length of the TFBSs is 15 bp and the consensus sequence, originally described as 5'-T-n11-A-3' (Goethals et al., 1992), has been extended to 5′-CTATA-n9-TATAG-3′ (Oliver et al., 2016). Based on DNaseI protection assays combined with structural analysis (Wang and Winans, 1995; Muraoka et al., 2003; Picossi et al., 2007), a model has been proposed for the molecular mechanism of the LysR-type transcription regulators when three TFBSs are present, which is the majority of the LysR-type intergenic organization (Figure 4A). The affinity of the TF for the first and last TFBSs is greater than for the second one. Then in absence of the co-inducer, the formation of the dimer imposes a large bending of the DNA from 50 to 100°. With the inducer, the dimer interacts with the second TFBS which releases the third TFBS and unbends the DNA of 9° up to 50°. From this conformation, the TF will interact with the α-subunit of the RNAP so that the transcription of the TG can start. This mechanism model was reinforced by DNA-binding studies performed on modified DNA sequences, and is known as the “sliding dimer” (Porrúa et al., 2007). To get insights into the molecular details of this model, several 3D structures were necessary and several examples will be described below.

The LysR-type genes code for proteins of around 330 amino acids. When searching for LysR transcription regulator in the PDB, it issues 87 entries corresponding to 27 different proteins, the majority corresponds to BenM from *Acinetobacter baylyi* or *sp*., DntR from *Burkholderia sp*. or *cepacia*, OxyR from *Vibrio vulnificus*, CysB from *Salmonella typhimurium*, TsaR from *Comamonas testosteroni* and AphB from *Vibrio vulnificus*. Their structure shows a HTH motif in the DBD at the N-terminus like the TetR family, and a regulatory domain (RD) receiving an inducer (Henikoff et al., 1988). A long helical linker separated the two domains. The HTH is the most conserved region and is used to identify the members of this family in genome analysis (Schell, 1993). In contrary to the TetR HTH motif, there are two additional β-strands between α3 and α4, a particular topology called winged-HTH. It also differs by the relative orientation of the three helices forming the DBD domain. Unlike the TetR family, the N-terminus of LTTR is localized at the dimer interface. As a consequence, all the helix axes are reverted as we can see by comparing Figure 3A with the DBDs on the ABS site of Figure 4C. The rest of the sequence is not very conserved except the C-terminal fragment of about 15 residues. Mutational analysis indicated implication in DNA binding or oligomerization (Schell et al., 1990; Bartowsky and Normark, 1991) which was partially confirmed with the first crystal structure of the RD of CysB from *Salmonella typhimurium*, the regulator of the cysteine regulon expression (1AL3, Tyrrell et al., 1997). The co-inducer binding domain is composed of two α/β Rossmann fold-like subdomains RD1 and RD2 (Figure 4B) with a long β-sheet in between. The RD architecture forms a bend where the co-inducer interacts. Punctual mutations introduced in the co-inducer binding site of CysB led to an uncontrolled activation phenotype in spite of a proper interaction with the TFBS according to gel-shift assays (Colyer and Kredich, 1996). The nature of the replacing residue was important since the mutant T149M is comparable to the wild type, whereas T149P shows only a 10% activity, which suggests that the conformational flexibility of the protein is required for the co-activator effect.

Several structures of the isolated RD domain were solved with a monomer or a dimer in the asymmetric unit of the crystal, but none of them reveals the functional mechanism by a tetramer. The first structure of a full-length LTTR corresponds to the one of CbnR from *Cupriavidus necator*, involved in the degradation of chlorocatechol converted from 3-chlorobenzoate, using cis,cis-muconate as inducer (1IXC and 1IZL: Muraoka et al., 2003). The structure shows a tetramer that can be considered as a dimer of dimer, composed of two types of subunits with different conformations (Figures 4B,C upper panel), either compact or extended forms. Within one dimer, the main interacting region corresponds to the helical linker α4 localized between the DBD and the RD. The two α4 helices bind in an anti-parallel manner, imposing the head-to-head orientation of both DBD interacting by their N-termini. In this architecture, the distance between the two α3 helices is compatible with an interaction with the major groove of the *B-*form DNA. The angle between α4 and the RD axis is about 50° in the compact form and 130° in the extended form. Contacts between two RD domains appear through the interaction of the tetramer, i.e., two LTTRs dimers. It has to be noticed that the so-formed RD dimer interface is similar to those described in the structures of the isolated domain from several LTTRs, such as BenM from *Acinetobacter baylyi* regulating aromatic compound degradation (2F8D: Ezezika et al., 2007a). The two dimers are properly superimposed without showing any hinge movement between RD1 and RD2, with the exception of the C-terminal α/β domain of RD1 that is more divergent. Because the last helix is localized in the continuity of the linker α4 in the extended form of CbnR (see Figure 4C upper panel), this swapping domain could be involved in the conformational changes necessary for proper function of this family of regulators. The global quaternary structure of CbnR is compatible with an interaction with two DNA binding sites on a bended DNA fragment, supporting that the crystal structure is biologically relevant. Among the different full-length structures solved later, the one of TsaR (3FXU and 3FZJ: Monferrer et al., 2010) from the soil bacteria *Comamonas testosteroni* brought interesting insights in the regulation mechanism of the LTTRs. TsaR regulates the degradation of paratoluenesulfonate (TSA), a commonly found industrial pollutant, that also induces the regulator transcription itself. The tetrameric structure solved in complex with TSA is flatter than CbnR and presents less contacts between the different RD domains as they swing almost perpendicular to the tetramer plane, which yields a different interface (Figures 4B,C lower panel). In this conformation, the hinge between the DBD and the RD reaches 153° whereas 130° was measured for CbnR. On the contrary, for the compact form, the angle of 50° is conserved. The distance between the two pairs of α3 helices of the DBD domain varies largely because of the surface convexity between two pairs of DBDs. It has been suggested that the open form structure of TsaR could represent the active tetramer whereas the more compact form of CnbR tetramer, through contacts between the third helix from the RD2 domains (α3-RD2) (see Figure 4B lower panel), would represent the inactive form. The transit to the active form could be induced by the binding of the TSA inducer in the cleft formed by two RD domains: in the structure of TsaR, the crossing β-sheet is broken in the middle when compared to CnbR. The hypothesis of a switch from a compact to an extended conformation of the tetramer once activated by the inducer in the RD cleft was confirmed by several LTTRs structures, i.e., ArgP regulating chromosome replication in *Mycobacterium tuberculosis* (Zhou et al., 2010), NdhR from *Synechocystis* involved in the control of carbon metabolism (Jiang et al., 2018), many of BenM (Ezezika et al., 2007b), and the SAXS experiments performed on DntR (Lerche et al., 2016). Nevertheless, the activation does not always depend on an inducer binding. For instance, a redox switch activates the oxidative stress regulator OxyR (Jo et al., 2015, 2017). Two cysteines (C199 and C208) from the helix α3-RD2 form a disulfide bond in the presence of H_2_O_2_, which results in the unfolding of the helix and subsequent conformation modifications.

Only three structures of LTTR were solved in complex with DNA [two of BenM (4IHT, 4IHS) and one of CbnR (5XXP)] and all of them concern only the N-terminus DBD, including the HTH and the α4 in order to stabilize the dimer (Alanazi et al., 2013; Koentjoro et al., 2018). As already mentioned, the antiparallel α4 helices coiled-coil and the N-terminus are at the proximity of the pseudo-palindromic DNA center. Helix α3 enters deeply in the DNA major groove and brings most of the specific contacts with the DNA bases. The wingled β-strand between the HTH and α4 makes contacts with the DNA minor groove, mainly through the phosphates, and one residue (R53) makes selective contact in BenM, which is absent in CbnR. Nevertheless, most of the selective residues are located in α3. Among these residues (A28, Q29, P30, P31, and R34 in CbnR), the remarkable mutation Q29A did not modify the interaction with DNA as proved by EMSA. Nevertheless, the mutant does not activate the transcription of the TG. This supports the importance of residues of the ABS site instead of the RBS. A plausible hypothesis is the involvement of Q29 in the RNA polymerase recruitment, since it is highly conserved among the LTTR family.

### The MarR family

The members of the Multiple Antibiotic Resistance Regulator (MarR) family are usually repressors found in bacteria and Achaea genomes (Wilkinson and Grove, 2006). They are mainly activated by sensors of environmental changes, like the presence of nutrients or toxins. Logically they often regulate genes coding for exporters of antibiotics, but they are also implicated in virulence, degradation processes, stress response and metabolic pathway (Alekshun and Levy, 1997; Perera and Grove, 2010). MarR family are also involved in aromatic compounds metabolism which is one of the attractive field in industrial research of renewable energy (Davis and Sello, 2010; Fuchs et al., 2011; Bugg and Rahmanpour, 2015; Kallscheuer et al., 2016; Grove, 2017). Due to the essential role, MarR family is widely spread in bacterial genomes, up to 24 in *Bacillus subtilis* according to UniProt data bank.

**Figure 5:**
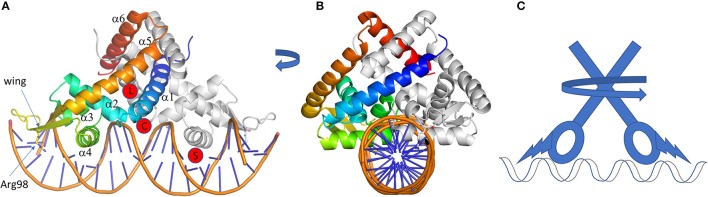
MarR family of regulators. **(A,B)** are two perpendicular views of a cartoon representation of a MarR family member, HcaR (pdb code 5BMZ). One monomer is gray, the other one is in rainbow. The conserved arginine 98 of the wing entering the DNA minor groove is represented in sticks. The different ligand-binding sites are indicated by red circles. They are labeled L for ligand, **(C)** for chloramphenicol from TcaR, and S for salicylate (see text for details). Whatever the activation mechanism (ligand binding, cysteine oxidation or pH sensing) the conformation change at the DBD is induced by a pincer movement of the helices at the dimer interface combined with a twist of the lower part of the protein, as modeled on the schematic representation in **(C)**. All the 3D structures are generated with PyMol (http://www.pymol.org; DeLano, 2009).

The MarR transcription regulators are small proteins of <150 residues containing a winged HTH domain at the N-terminus similar to the LTTR family but acting in a dimeric form like TetR family. About 120 structure entries are in the PDB and the first one (1JGS) corresponds to MarR regulator from *E. coli* (Alekshun et al., 2001). Compare to the other regulator families, the structure of MarR is quite simple, made of a DBD domain with an extension α-helix at each extremity (Figure 5). Those additional helices (α1 and α6) are the dimer interface. Besides, some MarR regulators present additional elements, for instance the regulator PcaV, involved in protocatechuate metabolism, an intermediary product of lignin degradation from *Streptomyces coelicolor*, possesses an additional β-strand between α2 and α3, forming a β-sheet wing (strands β1 and β2) (Davis et al., 2013). Another additional secondary structure is found in HucR from *Deinococcus radiodurans* (2fbk, Bordelon et al., 2006) involved in oxidative stress response to uric acid. HucR possesses an additional helix at its N-terminus that stabilizes the dimer in the absence of DNA by pinching the helices of the dimer interface.

TF from the MarR family recognizes one or two types of intergenic region among the different regulated genes. The TFBSs are 16 to 20 bp long and are not always perfectly palindromic (Martin and Rosner, 1995; Perera and Grove, 2010). Like the LysR family, the HTH motif (here α3 and α4) enters in the DNA major groove and the supplementary β-strand wing interacts with the minor groove. This wing extension is essential for DNA binding, especially the arginine of the loop connecting the β-strands (Kumarevel, 2012), which deeply enters the DNA minor groove (Figure 5A). The importance of this basic amino acid for the regulation mechanism was analyzed for the ST1710 regulator from *Sulfolobus tokodaii* involved in antibiotic resistance. Several basic residues from this loop were mutated into alanine (R90A), showing a decrease of binding affinity for the cognate DNA sequence by gel-mobility shift assays (Kumarevel et al., 2009). When two TFBSs are necessary for gene regulation, the binding position of two TFs is either on opposite side or adjacent on DNA depending on the intergenic length and of the size of the β-strands wing, even though a dissociation of the wing was reported in the structure of Rv2887 regulator from *Mycobacterium tuberculosis* in complex with two TFBSs of 30 bp (Gao et al., 2017).

Concerning the LBD, it is reduced to a smaller fold so that the “triangle cavity” described previously in the TetR family does not really exist, even if α5 and α6 seem to adopt a triangle-like shape (Figure 5). This structure is sufficient to create a cavity surrounded by helix α1 and sometimes a long loop between α1 and α2 like in ZitR, a zinc metalloregulator (Zhu et al., 2017). This cavity is a receiving platform for several kinds of molecules, like coumaric acid, ferulic acid, vanillin and 3,4-dihydroxybenzoic acid in the case of HcaR from *Acinetobacter*, a regulator of the hydroxycinnamate degradation pathway (Kim et al., 2016). The binding of a molecule into the cavity will rearrange the dimer interface conformation by modulating the distance between the two HTH domains and subsequently controlling the regulator release from the DNA. Nevertheless, structural modification of the regulator could be small for a large group of MarR regulators, like in SlyA, a virulence regulator from *Salmonella* (Dolan et al., 2011). The apo-form and ligand-bound structures are already in a conformation favorable to *B*-DNA interaction, with a distance of around 30 Å between the two DNA recognition helices. In this case, the presence of the inducer simply add stability to the regulator-DNA complex as demonstrated on MexR by thermal unfolding experiment and surface plasmon resonance (Andrésen et al., 2010). This is also supported by the structure of PcaV (4G9Y and 4FHT; Davis et al., 2013) showing the importance of an arginine (R15) in the functional mechanism (Figure 5A). By comparing the structure of the apo-form and the regulator complex with the natural ligand 3,4-dihydroxybenzoic acid, the R15 occupies the binding site in the absence of ligand. It is then pushed away by the ligand and forms hydrogen bonds with residues localized in α2, α3, α3-α4 loop, α1 of the other monomer and the ligand itself. This pulls α1 by 10° toward the second monomer with an allosteric effect on the DBD orientation and a large movement of the β-sheet wing. To sum up, R15 and the ligand make a stable bridge between the DBD and the dimer interface by many hydrogen bonds. This specific mechanism was also described for NadR, the Neidderial Adhesin NadA repressor from *Neisseria meningitidis* studied by HDX-MS and molecular dynamic (Brier et al., 2012). Other binding sites were suggested for this family. In the case of the monomeric MarR (1JGS), two molecules of sodium salicylate bind on each side of the HTH-α4 of the DBD domain (Figure 5A). This compound is an inhibitor of MarR that will activate *marA* gene transcription (Cohen et al., 1993). It suggests the possibility of a regulation mechanism directly at the DBD site. Several other possible interacting sites were described, based on the structures of TcaR solved with different compounds (4EJT, 4EJU, 4EJV, 4EJW, Chang et al., 2010, 2013). For instance, kanamycin binds to similar sites as the ligand of PcaV, but chloramphenicol binds to an unusual large cavity below the dimer interface (see C in Figure 5A), as well as a second site close to the α4. The latter is similar to that of salicylate in MarR or MTH313, a MarR homolog from *Methanobacterium thermoautotrophicum* (Saridakis et al., 2008).

Nevertheless, ligand binding is not the only activation mode of MarR family regulators. Some of them are sensitive to oxidative stress like AbfR from *Staphylococcus epidermidis* (Liu et al., 2017). The monomer possesses two cysteines, each one in the terminal helices (α1 and α6). The regulator binds to DNA in a reduced state. Under oxidative environment, sulfenic acid intermediates catalyse disulfide-bridge formation of the cysteines, which results in a large movement of the two monomers and destabilizes DNA interaction. The same regulation mechanism is also found in OhrR, the regulator of a peroxidase that reduces organic hydroperoxides to alcohols (Newberry et al., 2007). Upon oxidation by hydroperoxides, helix α5 of OhrR is locally unfolded which brings in close proximity the two cysteines to form the disulfide-bond responsible for a rigid-body rotation of the winged-HTH and then DNA release. Another regulation mechanism that depends on the pH was described for HucR regulator (Deochand et al., 2016). As shown by circular dichroism study at different pH, the N-terminal helices from each monomer interact by H51 stacking which protonation results in a molten globule intermediate with a low affinity for DNA. This explains the necessity for an additional helix at N-terminus to maintain the 3D structure of HucR during the conformation changes.

### The AraC/XylS family

The *t*ranscription *r*egulators of *A*raC *f* amily (AFTRs) are generally activators found in all bacterial genomes, except in archaebacteria (Gallegos et al., 1997). Like most of activators, the main TFBS is located around the−35 region of the promoter, which eases direct interaction with the RNAP (Ptashne and Gann, 1997). The members of the AFTR group are involved in pathogenesis, virulence and environment sensing, responding to oxidative stress, pH, temperature and antibiotics. They are also highly implicated in the regulation of essential metabolism pathways of the carbons, such as sugars, amino acids, alcohols or herbicides degradation (Gallegos et al., 1997; Egan, 2002; Ibarra et al., 2008).

The regulators of this family are generally 300 residues long, with some exceptions such as MarA or SoxS from *E. coli* which are reduced to the sole DBD domain (Rhee et al., 1998). When searching “AraC transcription” in the PDB, only 22 entries are listed which is low compared to the other families. An explanation was published in an essay of Schleif and his group who investigated the AraC regulation mechanism (Schleif, 2003). Because AraC interacts with more than 40 bp, it must be partially unfolded in the absence of DNA to reduce the binding energy for a reversible interaction. Thus, the intrinsically disordered state of this regulator family makes them difficult to handle (Schleif, 2010). Nevertheless, with the constant progress in protein expression and purification methods, several structures were obtained for more than ten different members of the family. Most of the ATFRs are made of two domains: a response domain (RD) involved in protein dimerization and a conserved DBD of around 100 residues. The HTH motif interacts with the DNA in a similar way to the TetR family with one helix entering the DNA major groove. But unlike TetR, there is no wing interacting with the minor groove and one monomer carries two HTH separated by a long linking-helix. When both HTH interact with two contiguous DNA major grooves (Rhee et al., 1998), the DNA is bended by ≈35° (Martin and Rosner, 2001). Besides, some proteins, such as the transcription factor Rob involved in antibiotic resistance and organic solvent tolerance in *E. coli*, makes direct contacts with only one DNA major groove with the first HTH, whereas the second interacts with the RNAP (Bhende and Egan, 2000). The tandem HTHs are generally at the C-terminus but in Rob regulator (PDB code 1D5Y), the HTH motifs are localized at the N-terminus. Even though the interacting configuration of the AraC family is quite specific, HTH motifs are poised from either side of the DNA fragment (Kwon et al., 2000) in a comparable manner to the QacR sub-group of TetR. Affinity measurements and DNA foot-printing experiments show that AraC recognizes DNA sequences as a tandem or inverted repeat orientation with different affinities (Carra and Schleif, 1993; Reeder and Schleif, 1993).

**Figure 6:**
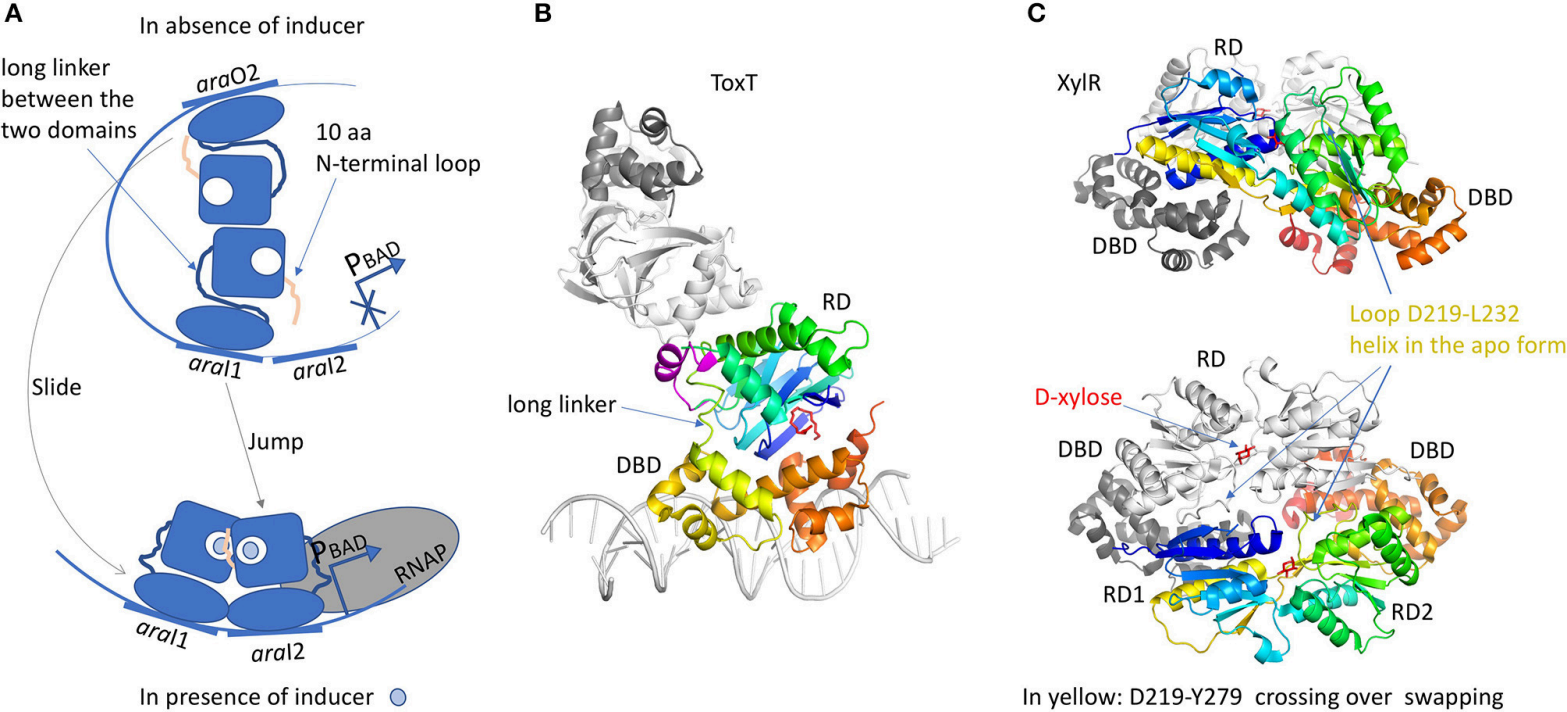
AraC family of regulators. **(A)** Illustration of the “DNA-looping” and “light-switch” models as described in the text. The proximal *ara*I1 and the distal *ara*O2 are separated by around 200 bp. The addition of arabinose will trigger the movement of the N-terminal loop that will close the arabinose-binding site and release arabinose from the DBD, giving more flexibility to the protein (“light switch” mechanism). One dimer will shift from *ara*I1 to *ara*I2 and the other one from *ara*O2 to *ara*I1, together with a modification of the dimer conformation (“DNA-looping” mechanism). The new dimer will be closer to RNAP and then will be able to induce the *araBAD* promoter. **(B)** Structure of ToxT (3GBG) colored in rainbow. The second monomer in gray was generated by crystallographic symmetry in order to illustrate a possible dimer formation via the RD domain. The DNA fragment was modeled according to MarA structure (1BL0). Palmitoleic acid is in red. The small transition-helix is modeled in magenta from 4MLO structure, in close proximity to the loop linking the RD to the DBD. **(C)** Structure of a dimer of XylR (4FE4, 4FE7) showing an AraC DBD domain and a LacI-RD domain. One monomer is colored in rainbow and the other one in gray. The swapping domain D219-Y270 is colored in yellow and orange. This dimer illustrates a possible “proximal dimer” conformation of the DNA-looping mechanism. All the 3D structures are generated with PyMol (http://www.pymol.org; DeLano, 2009).

The RD domain is not conserved across the AraC family and it has very different functions. Both RD and DBD domains are independently associated by a long linker. A chimeric construct of the AraC-RD with the LexA-DBD from the LexA repressor (Bustos and Schleif, 1993) results in a protein that was able to dimerize and to repress *lexA* operator in response to L-arabinose. In spite of more than 20 years of work, the full-length structure of AraC is still unsolved. Nevertheless, crystal structures of the RD domain were solved with and without the arabinose ligand (Soisson et al., 1997; Weldon et al., 2007). The domain is a jellyroll that ended by a helix coiled-coil. RD dimer is found in the asymmetric unit only in the presence of arabinose. The dimer interface involves hydrophobic residues from the coiled-coil motif and an additional helix from the region between the jellyroll and the coiled-coil. The arabinose binds in a cavity formed by the jellyroll and locked by N-terminal loop of 10 residues. Without arabinose, this loop was not visible in the electron density. Genetic, biochemical and biophysical characterizations of AraC brought hypothesis on the regulation mechanism by the N-terminal loop (Carra and Schleif, 1993). In *E. coli*, L-arabinose is involved in the regulation of four operons, *araBAD, araE, araFGH* and *araC*. AraC represses the expression of *araBAD* and *araC* promoters by binding to two different DNA half sites separated by around 200 bp, the proximal site *araI1* and the distal site *araO2*, leading to the formation of a DNA loop (Figure 6A). In this configuration, the N-terminal loop makes contacts with the DBD, constraining the monomer in a compact structure. Arabinose binding results in the release of this loop by a “light switch” mechanism, allowing the RDs to form a totally different dimer. Then the DBD shifts from *araO2*/*araI1* to *araI1*/*araI2* sites, closer to the RNAP and the induction of the *araBAD* promoter. This mechanism is found in several regulators like ToxT from *Vibrio cholerae* (Lowden et al., 2010) or RegA from *Citrobacter rodentium* (Yang et al., 2009, 2011) respectively regulated by fatty acids or bicarbonate. The structure of the full-length protein ToxT from *Vibrio cholerae* was solved in the absence of DNA (3GBG; Lowden et al., 2010; Figure 6B). The RD domain is very similar to that of the AraC but without the N-terminal loop. Instead, Li et al. (2016) revealed a small α-helix (D101 to E110) in proximity of the RD to DBD linker. Mutational analysis in ToxT of Met103, Arg105 or Asn106 showed a threefold activation increase of the *ctxA* promoter, pointing out the important role of this small α-helix (Childers et al., 2007). It could play the same role as the N-terminus loop of AraC by controlling protein flexibility. Another regulator of the AraC family was solved full-length: XylR (4FE4, 4FE7; Ni et al., 2013; Figure 6C). It is activated in *E. coli* in the absence of glucose, in order to use D-xylose as an alternative carbon source (Brückner and Titgemeyer, 2002). From structural comparison, the C-terminal DBD domain is very similar to that of AraC, but not the N-terminal RD domains. The XylR-RD domain looks like the periplasmic binding-protein of PurR from the LacI/GalR regulator family. It is composed of two α/β sub-domains linked by a small loop. The ligand-binding cavity is localized at the interface between the two α/β sub-domains. At the beginning of the swapping region between the RD and DBD domains, a flexible loop (D219-L232) in the complex structure with D-xylose changes into α-helix in the apo-form, which modulates the binding affinity of the DBD toward the cognate DNA operator. XylR forms antiparallel dimers orienting the DBD domains in a head-to-head manner. Because XylR has to interact with two distant operator sites, a DNA loop must be formed by the DBD dimer, as demonstrated by AFM studies performed on XylR dimer in complex with a 500 bp DNA fragment. Note that unlike AraC, the DNA loop is formed in presence of the inducer ligand D-xylose. Nevertheless, there is a cross talk between the two regulatory mechanisms since AraC binds a DNA region containing the XylR promoter. It gives the possibility to activate one or the other sugar-producing pathway according to the available source of carbon.

All of the AFTRs does not respond to this simple mechanism. For example, InvF from *S. enterica serovar Typhimurium* is not able to activate virulence genes without the chaperone protein SicA (Darwin and Miller, 2000). It is suggested that InvF would function as a monomer but associated with SicA. MarA (Rhee et al., 1998; Gillette et al., 2000) and SoxS (Griffith and Wolf, 2002) involved in response to oxidative stress are small proteins of around 100 amino acids, formed by HTH domain only, thus devoid of responsive domain for a signaling ligand. They act as monomers and their regulation depends on the position of the TFBS toward the RNAP.

The tight regulation mechanism of AraC has inspired biotechnology developments and it is largely used as bacterial expression systems for recombinant protein expression (Brautaset et al., 2009).

### The one-component regulators of *P. aeruginosa* RND efflux pumps

As mentioned in the introduction, very few structural information is available on *P. aeruginosa* regulators, with only three solved structures to date: MexR, NalD and MexZ. Two of them are involved in the regulation of the MexAB-OprM pump which is considered as constitutive, although mutations in either *mexR, nalC* or *nalD* cause an over-expression of the pump (Boutoille et al., 2004; Sobel et al., 2005). MexR is the primary regulator of MexAB-OprM; it belongs to the MarR family and binds to its own promoter and that of the *mexAB-oprM* operon. One MexR regulatory pathway involves the binding of ArmR, a polypeptide of 53 amino acids, which expression is controlled by NalC, another TetR repressor. The structure of NalC has not been solved yet but it shows 31% of similarity with the N-terminal half of MLR_4833 from *Mesorhizobium loti* (3BHQ), corresponding to a canonical DBD of the TetR family. The LBD structure is not known but pentachlorophenol and other chlorinated phenol molecules have been identified as NalC signaling inducer (Muller et al., 2007; Ghosh et al., 2011). Several structures of MexR were solved: in the apo-form (1LNW, Lim et al., 2002), in complex with the C-terminal part of ArmR (3ECH, Wilke et al., 2008) and a clinical mutant (R21W) that induces overexpression of the pump (4ZZL, Anandapadamanaban et al., 2016). In addition, another apo-form structure was solved upon oxidation of the cysteines (3MEX, Chen et al., 2008), highlighting a new mechanism of regulation by inter-monomer disulfide bond (Chen et al., 2010). The comparison between the four different structures gives some information on the induced conformational changes necessary for the regulation. In the MexR/ArmR complex, ArmR binds into the classical MarR cavity with an oligomeric ratio of 1:2. The C-terminus enters deeply in the protein toward the cavity of the second monomer, labeled as “C” on Figure 5. In the mutant R21W structure, the dimer is more packed with a more constricted ligand cavity: the tryptophan mutant is stacked in between four prolines (P37 and P38 from each monomer) at the position normally occupied by G49 of ArmR (Figure 5C). This will cause the closure of the pincer formed by helices α1 and α6, and consequently will increase the distance between the two β-wings. The dimer does not fit anymore to a *B-*DNA conformation, which releases the repression and promotes *Pseudomonas* antibiotic resistance by MexAB-OprM efflux. The structure of the oxidized MexR is comparable to that of mutant R21W despite the 16 Å displacement of the α3-α4 loop resulting in the disulfide bond formation between C62 and C30 from each monomer. The distance between the two helices α4 of the wilt-type dimer (29 Å) is more suitable for tandem DNA major grooves interaction (34 Å) compared to the mutant (23.5 Å). These structures have brought important information to understand the regulation mechanisms of MexR.

Another regulator of known structure is NalD, a secondary regulator of MexAB-OprM transcription (Morita et al., 2006). NalD is a TetR repressor that recognizes a TFBS upstream the operon *mexAB-OprM*. The NalD structure was solved in its apo-form (Chen et al., 2016) and is very similar to that of TtgR, the regulator of TtgABC efflux in *Pseudomonas putida*, which has a less folded α4 helix. It was proved that NalD is able to bind to novobiocin in a similar pocket to that of TtgR, resulting in resistance increase of *P. aeruginosa* strains.

The repressor NfxB of the MexCD-OprJ efflux pump was first classified in the LacI/GalR family, but the closest sequence homology turns to be the TetR-like regulator LFRR from *Mycobacterium smegmastis*, especially the DBD domain. Besides, MexL also belongs to the TetR family, with sequence identity alignment coverage of 93%. It shares 46.5% sequence identity with the DBD domain of NalD, which suggests a possible competition for the same TFBS. Another TetR repressor is the primary repressor MexZ of the MexXY-OprM efflux pump, which selectively transports aminoglycosides. The structure of MexZ was solved in its apo-form (Alguel et al., 2010). It presents a classical TetR structure, with a partially unfolded α4 helix. At present, the inducer molecule of MexZ is not known. A novel protein partner ArmZ, classified as a RNA-ligase, is suggested to sequestrate MexZ which releases the repression of *mexXY* (Hay et al., 2013). The gene of MexZ is the most frequently mutated in *P. aeruginosa* strains from cystic fibrosis patients (Smith et al., 2006). Most of the mutations are found in the DBD or strategic positions such as the dimer interface. One mutation has been reported on the surface of the helix α7 (L128M, see Figure 3; Guénard et al., 2014), which suggests α7 as part of the recognition site of ArmZ.

Finally, an activator regulates the efflux pump MexEF-OprN this time: MexT, which belongs to the LysR family (Fetar et al., 2011). MexT acts as a primary regulator of the pump together with the repressor of *oprD* porin. A redox mechanism seems to regulate MexT through *mexS* gene which codes for an oxydoreductase that upregulates MexEF-OprN (Morita et al., 2015; Richardot et al., 2016). We modeled the structure of MexT based on that of the DntR regulator from *Burkholderia* and localized in the RD domain the only cysteine that could possibly react to the ROS (Reactive Oxygen Species). Nevertheless, this cysteine can hardly form a disulfide-bond within a dimer of MexT according to the homology model structure. Recently a secondary activator of MexEF-OprN was described to upregulate MexEF-OprN through MexS and MexT (Juarez et al., 2018): it is named CmrA for Chloramphenicol Resistance Activator (Juarez et al., 2017). This regulator belongs to the AraC family and presents several cysteines, which is interesting in the context of the redox regulation mechanism.

## Conclusion

As a multidrug-resistant pathogen, *Pseudomonas aeruginosa* possesses many RND efflux pumps. But some of them are functionally redundant, which is *a priori* not needful in term of biological evolution. Surprisingly, efflux pumps that transport similar molecules are not regulated by the same transcriptional systems. This certainly reflects the need to a prompt reactivity of the bacteria upon environment modification. All the 3D structures of the different regulators solved from different bacteria brought complementary informations to genetic, biochemical and biophysical data, in particular the crystal structures gave important insights in the comprehension of the main regulatory mechanisms. Nevertheless, with the intention of doing specific drug-design, high-resolution structures of regulators from *P. aeruginosa* are still necessary. Recent structures of the virulence factor regulator MvfR, member of the LysR family from *P. aeruginosa*, solved in complex with inducer and inhibitor, illustrate the interest of the structural approach (Kitao et al., 2018). Both molecules bind within the same cavity with subtle interaction differences that are the keystone of the regulation mechanism. Thus, the knowledge of the 3D structures of each specific regulator is mandatory to develop new and specific drugs. From the complex signaling regulation of RND pumps expression illustrated on Figure 1, it is clear that there is a real lack of information concerning the structures of *P. aeruginosa* regulators. As this bacterium belongs to the group of the most problematic clinical pathogens, structural study of the regulators of *P. aeruginosa* are urgently needed.

## Author contributions

All authors listed have made a substantial, direct and intellectual contribution to the work, and approved it for publication.

### Conflict of interest statement

The authors declare that the research was conducted in the absence of any commercial or financial relationships that could be construed as a potential conflict of interest.

## Abbreviations

ABS: Activation binding site
ABC: ATP-binding cassette
CA: Catalytic and ATP binding domain
DBD: DNA-Binding Domain
DHp: Dimerization and Histidine phosphotransfer domain
GAF: cGMP-specific phosphodiesterases, Adenylyl cyclases, FhlA domain
HAE1: hydrophobe/amphiphile efflux 1
HAMP: Histidine kinases, Adenyl cyclases, Methylaccepting proteins, Phosphatases domains
HK: Histidine Kinase domain
HTH: Helix-Turn-Helix
LBD: Ligand binding domain
LTTR: LysR-Type Transcription Regulators
MATE: Multidrug and toxic compound extrusion
MDR: Multidrug resistance
MES: Multidrug efflux systems
MFS: Major facilitator superfamily
PAS: Per Arnt Sim domain
PDB: Protein data bank
RBS: Regulatory binding site
RD: Regulatory domain
REC: N-terminal Receiver domain
RND: Resistance-nodulation-cell division
ROS: Reactive oxygen species
RR: Response Regulator
SD: Sensor Domain
SK: Sensor Kinase receptor
SMR: Small multidrug resistance
TCS: Two-Component System
TetR: Tetracycline repressor
TF: Transcription factor
TFBS: Transcription factor binding site
TG: Target gene
TM: Trans-Membrane domain
TSA: paratoluene sulfonate.
